# Polymer Prosthetic Hand with Finger Copies for Persons with Congenital Defects or After Amputation Using 3D Printing Technology

**DOI:** 10.3390/polym17141983

**Published:** 2025-07-19

**Authors:** Anna Włodarczyk-Fligier, Magdalena Polok-Rubiniec, Aneta Kania, Sebastian Jakubik, Jakub Painta, Justyna Ryś, Jakub Wieczorek, Marta Marianek, Agata Ociepka, Mikołaj Micuła, Jakub Osuch

**Affiliations:** 1 Department of Engineering Materials and Biomaterials, Silesian University of Technology, Konarskiego 18a St., 44-100 Gliwice, Poland; magdalena.polok-rubiniec@polsl.pl (M.P.-R.); aneta.kania@polsl.pl (A.K.); 2 Faculty of Mechanical Engineering, Silesian University of Technology, Konarskiego 18a St., 44-100 Gliwice, Poland; sj307075@student.polsl.pl (S.J.); jp307643@student.polsl.pl (J.P.); jr307675@student.polsl.pl (J.R.); jw307697@student.polsl.pl (J.W.); mm307631@student.polsl.pl (M.M.); ao307638@student.polsl.pl (A.O.); 3 Faculty of Electrical Engineering, Silesian University of Technology, Bolesława Krzywoustego 2 St., 44-100 Gliwice, Poland; mm311135@student.polsl.pl; 4 Faculty of Automatic Control, Electronics and Computer Science, Silesian University of Technology, Akademicka 16 St., 44-100 Gliwice, Poland; jo310975@student.polsl.pl

**Keywords:** biomaterials, PLA, prosthetic hand, 3D printing, Arduino

## Abstract

The research presented in this paper focuses on the utilization of 3D printing technology in the design and manufacture of a prosthetic hand, equipped with a digit replicator. The subject of this study was a young man who had undergone the amputation of two fingers on his right hand. The electronic control of the movement of the finger copy was developed using Arduino language. A concept and outline drawings were developed in ProCreate. Three-dimensional scan of the hand and forearm was made using an EinScan PRO HD SHINING 3D scanner. Using CAD software—Autodesk Inventor and Autodesk Meshmixer, the prosthesis was designed. Printing was carried out on a 3D printer of the i3 MK3 and MK3+ series using a PLA (polylactic acid) filament. It was determined that PLA is an optimal material for printing, as it is considered to be safe for future patients’ skin. Work on the electronic circuitry started in Autodesk TinkerCad simulation software, allowing the code to be verified and ensuring the safety of the control system. The prosthesis’s design demonstrates the potential to reach as many people in need as possible by using readily available, low-cost, and easy-to-use components.

## 1. Introduction

People who have had a limb amputated often experience psychological problems such as grief, anxiety, and depression, as well as difficulty accepting their new situation. Amputation is a significant life change that can lead to disruption to daily life, as well as a loss of security and self-esteem. Thanks to advanced technologies, prostheses can fully or partially restore people’s mobility and freedom of movement so that they can perform any activity and improve their functioning in daily life [1,2,3,4]. The disadvantage of prostheses is that they must be individually adapted to the specific needs of the patient depending on congenital defects or mechanical damage. The personalization of the prosthesis affects its cost by using appropriate materials that differ in price, which ultimately affects the cost of the finished product [5]. The prices of prostheses can range from several thousand to tens of thousands of Polish złoty. Therefore, choosing the right prosthetic material is one of the most important tasks for specialists [6,7,8]. Another problem is that a significant portion of the materials oxidizes or corrodes as a result of interaction with human tissues or body fluids. The main materials used to manufacture prostheses using 3D printing are PLA [7,9], PET-G [10], ABS [11], and silicone [12].

The patient’s possession of a prosthesis, regardless of its type, fulfils many tasks. One important task is to minimize the risk of pain that may result from overloading the dominant side. When one limb is amputated or missing, the other limb takes over to perform all the activities that were previously performed by both limbs. As a result, one arm is overloaded, as it is the one that performs twice as many activities as before.

Upper limb prostheses are prepared by taking into account the physical parameters of the person, as well as expectations regarding the functionality of the prosthesis. Prostheses are divided into passive (cosmetic), mechanical (kinetic or functional), and hybrid prostheses, which directly control the muscles to enable movement. A cosmetic upper limb prosthesis should mirror the lost limb, although it does not have functional functions such as internal mechanisms. The main purpose of this type of prosthesis is to visually camouflage the lost limb and therefore can be used to a limited extent for daily activities [2,13].

A functional upper limb prosthesis has moving parts that are designed to restore mobility and the basic functions of the lost limb using preprogrammed mechanically controlled tools. Although a functional prosthesis is heavier than a cosmetic prosthesis and requires more handling, it allows a high degree of independence in the patient’s daily functioning. Additionally, based on the level of amputation, prostheses can be divided into [1,3,13] partial prostheses of the hand, forearm, upper arm, and shoulder joint extensions.

The last group of upper limb prostheses are myoelectric (bionic) prostheses. In these prostheses, the hand is powered by batteries that must be regularly recharged. The function of this group of prostheses is based on the muscles inducing contractions that cause impulses to be processed by electrodes placed in the prostheses. As a result, electric motors located in the hand are activated, which, in turn, causes the hand to move [14].

The selection of an upper limb prosthesis depends on many factors. One of these is the individual question of how functional the prosthesis should be, what range of movement it should allow the patient, and whether it should only visually reproduce the lost body part. Another important factor is how much money the patient can afford. It is important that the prosthesis is made to the patient’s individual specifications, as this will improve function [14].

The 3D printing process creates three-dimensional objects based on a model made in computer-aided design (CAD) software (student version) [15,16]. Digital models of individual denture components are manufactured layer by layer using various materials, including plastics, metals, and composites [17]. Prototyping is incremental manufacturing and differs from traditional manufacturing methods such as machining or casting. Professional 3D printing technology is characterized by simplicity and speed of printing unlike other methods, making it a key manufacturing method in various industries such as prosthetics [6,17,18]. The flexibility of 3D printing also allows for the combination of different materials in the same prosthesis. To illustrate this point, consider a prosthetic hand, such as a robotic hand. In such a scenario, rigid components may be fabricated from ABS, thereby providing structure and support, while areas requiring movement or skin contact may be printed with TPU, thus ensuring comfort and flexibility [5].

Plastic production represents a significant ecological challenge, attributable to excessive production, inadequate waste management, and the detrimental impact on land and water ecosystems [19]. As a class of particulate matter, microplastics are a cause for significant concern due to their capacity to penetrate biological organisms, thereby exerting deleterious effects on health and ecological systems [20]. The increasing emphasis on environmental protection has led to a shift in the use of renewable and biodegradable plastics to replace chemically derived plastics [9,19]. Polylactide (PLA) is one of the most commonly used polymers in 3D printing technology. Properties such as its low price, availability, and density of 1.24–1.30 g/cm^3^ contribute to its widespread use. Recent articles have presented PLA synthesis and its applications [9,21]. In the study [21], Hussain et al. present a comprehensive overview of the most important aspects of PLA, encompassing its synthesis, properties, biodegradation mechanism, and, finally, its applications in various sectors of the economy. The authors devote a significant portion of the text to the utilization of PLA in the field of medicine. They corroborate the assertion that the optimization of processing techniques and the judicious selection of additives are instrumental in attaining the desired properties of PLA. Dana et al. [9] present a comprehensive analysis of the diverse syntheses of PLA, exploring the multifaceted properties that define this material and its significant applications [9]. Despite its high strength, it is brittle mainly due to its low elasticity. Under sudden loads or impacts, it has a tendency to easily crack. Another disadvantage is its low temperature resistance; it softens at temperatures as low as approx. 60 °C, making it unsuitable for applications requiring heat resistance. Due to its biocompatibility and low toxicity, it is used in medical applications but requires a number of product safety standards to be met. PLA does not cause any side effects when decomposing in the body as happens with other materials, which is why it is used, among other things, for the manufacture of various types of implants. Implants that are in the body decompose, so there is no need to remove the implant from the body as is the case with some metal implants. Furthermore, it does not emit any harmful fumes during printing, which classifies it as a safe material [1,7,16,22,23,24].

This article describes the design and fabrication of a prototype prosthetic hand made from PLA for a male patient who underwent a double amputation of his right hand. The prosthesis incorporates an electronic control system that utilizes an Arduino microcontroller to control the fingers, employing TinkerCad software (online version) to facilitate system modelling without the necessity for physical components. The prosthesis was developed with comfort, safety, and aesthetics in mind, while maintaining a low price, which should not exceed PLN 1500, to ensure its availability to a wider audience.

## 2. Materials and Methods

The values of the parameters presented in the research were ascertained through a series of trial experiments, leading to the selection of the most optimal values for the design of the prosthesis.

In order to evaluate existing solutions, a concept and contour drawings of the prosthesis were made in ProCreate program version 5.3.15. Then, a 3D scan of the hand and forearm was made using an EinScan PRO HD SHINING 3D scanner (Keyence, Mechelen, Belgium) in manual mode. This device can capture up to 3,000,000 points per second. The volumetric accuracy with marker setting is up to 0.045 + 0.3 mm/m. The distance between points is 0.2–3 mm, the scanning accuracy is up to 0.04 mm, and the maximum field of view is 310 × 240 mm.

Next, the prosthesis was designed using CAD software—Autodesk Inventor 2025 and Autodesk Meshmixer version 3.5.0 (Autodesk, San Rafae, CA, USA) [16]. In this way, the shape and appearance of the prosthesis can be optimized. In order to perform a correct scan, the operation was performed in a darkened room. An important aspect was to immobilize the patient’s hand and forearm to homogenize the scan. The patient’s hand and forearm had to be scanned three times, precisely from each side, in order to process the FEM mesh.

The internal and external part of the prosthesis, which will adhere to the patient’s hand, was designed using the honeycomb method with a 25% filling [7]. The honeycomb structure also provided a lower weight for this part of the prosthesis. MESHMIXER program version 3.5.0 selected the wall thickness and size of the prosthesis holes. The rounding of the prosthesis edges was introduced to reduce irritation and discomfort when wearing.

The finger model was divided into several elements to facilitate assembly. The modeling process also included holes on the phalange joints, to which pins were assembled at a later stage. Efforts were made to accurately reproduce the smooth movement of functional fingers. The freeform shape function and surface modeling were used to reproduce human fingers. The connection of the phalanges with the prosthesis and boxes allowed for precise visualization of the prosthesis. Then, the prosthesis model was transferred to Prusa Slicer 2.8.1 software. The prosthesis was printed on a 3D printer—i3 MK3S & MK3S+ (Prusa Research, Prague, Czech Republic) using a PLA (polylactic acid) filament due to its safety for use on the skin of the future patient [21,22]. Work on the electronic circuits began in Autodesk TinkerCad simulation software (Autodesk, California, USA), which allowed for code verification and ensured the safety of the control system.

TinkerCad program is intuitive and has a component database with sample circuits showing how to assemble individual elements of the prosthesis. The program allows connecting components to a contact board. The following components were used: Arduino Nano Every; (Arduino, Scarmagno, Italy) two servos with 180° rotation, which can be powered by 5 V; two 73 × 6.3 mm deflection sensors; and a battery. The main assumptions that were made when writing the program were simplicity and full functionality of the prosthesis. The sensors were connected to the analog pin and the pins to the servos for proper control. During preparation, two objects were developed that corresponded to two engines. In the next stage, the deflection angle was converted to the rotation angle in order to regulate the finger bending angle via a cable. After switching on the system, the servos set themselves to the 0° position. Finally, a time delay was added to ensure that the system smoothly worked.

## 3. Results and Discussion

### 3.1. Conceptual Phase—Assessment of Potential Solutions

The human hand is a ‘biological machine’ that facilitates a range of precise and delicate activities, including grasping and lifting. The loss of such upper limbs can result in a significant disruption to an individual’s ability to perform daily activities, which may consequently lead to the development of emotional and psychological problems [25].

A survey of the literature on artificial hands and fingers reveals the existence of information pertaining to the design of artificial fingers, in which mechanics are combined with electronics, with consideration given to touch sensors [26,27]. Moreover, there are works describing the use of new materials for hand and finger prostheses, sensors, and manufacturing processes using, among others, 3D technology [4,5,25,26,27]. In the work [25], Siegel et al. conducted a comparative analysis of the grasping capabilities of three commercially available prosthetic hands and three 3D-printed prosthetic hands. The investigation revealed disparities in grip performance, which may be attributable to inadequately planned design processes or substandard printing quality.

Taking the above into account, the concept of designing and manufacturing, using 3D technology, a hand prosthesis made of light PLA polymer was created. The individual concerned was a 24-year-old man who had sustained a loss of fingers 4 and 5 of his right hand.

**Figure 1:**
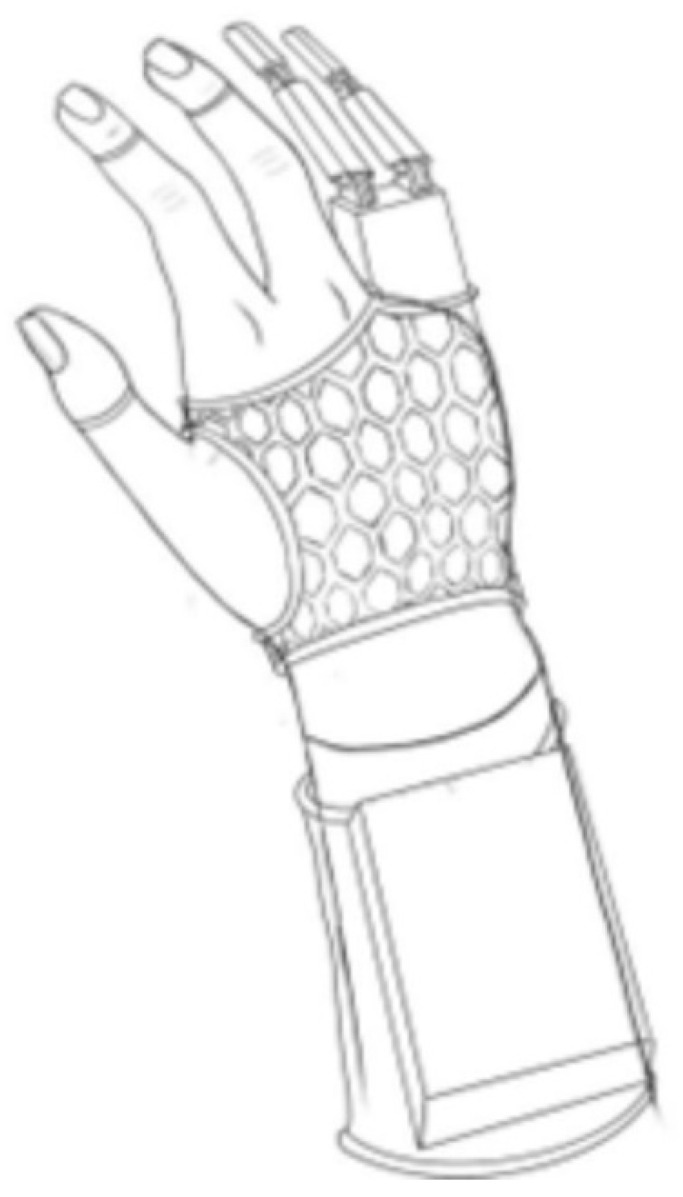
Schematic drawing showing a first concept of a control module on the forearm with a microcontroller placed in the housing.

**Figure 2:**
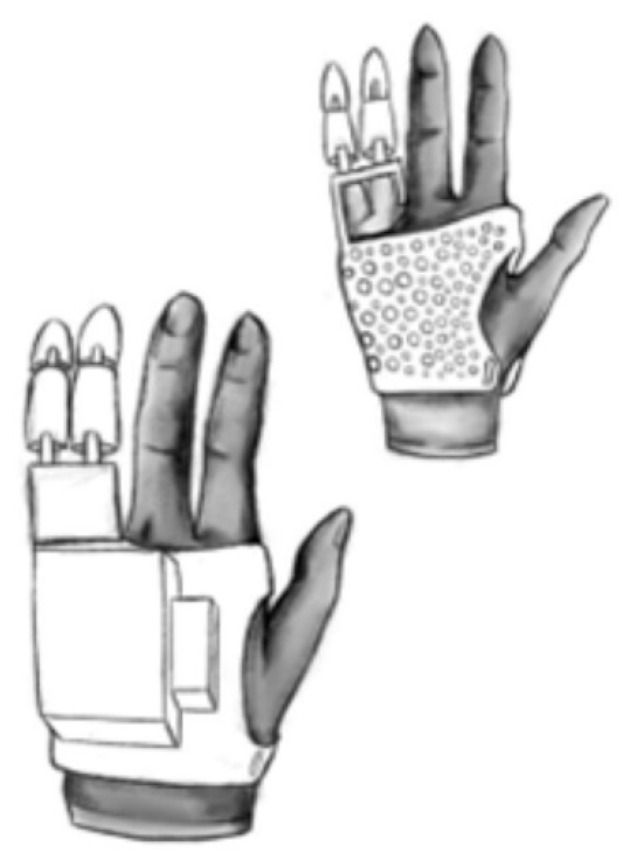
Schematic drawing showing a second concept of a control module on top of the hand, with a microcontroller placed in a box.

In the first stage of prosthesis design, an attempt was made to find the most effective and practical solutions. To this end, outline drawings of the prosthesis were made. Two alternative concepts were considered (Figure 1 and Figure 2).

The first version (Figure 1) assumed that the control module, containing the microcontroller and power source, would be placed on the user’s forearm, hidden in a box printed from the PLA filament on a 3D printer. On the hand is a lightweight, honeycomb-like structure, also printed from the same polymer. A billiard glove is placed under the mesh structure to prevent abrasion of the patient’s hand skin due to the rigidity of the structure. In this concept, the servos were placed on the top of the hand, while the deflection sensors were placed under the billiard glove.

In the second concept (Figure 2) under consideration, the component layout was changed—all power and control components were placed on a box located on the top of the hand, eliminating the need to run wires and mechanisms from the forearm up to the hand. The microcontroller was reduced in size, and all controls were placed in a box. The design is based on a lightweight case printed from PLA, providing breathability and reducing the overall weight of the prosthesis [9]. This concept also uses a billiard glove to avoid abrasions to the skin of the hand and fingers.

### 3.2. Three-Dimensional Scanning

**Figure 3:**
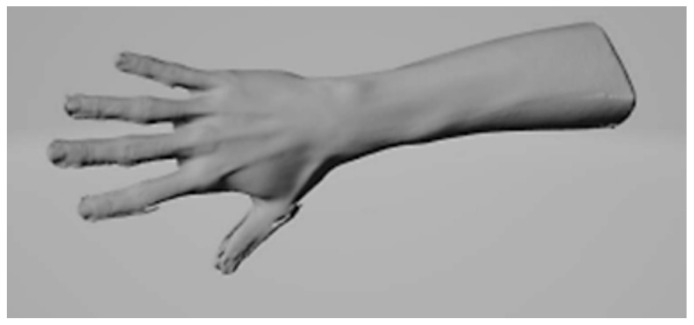
Scan of the hand and part of the forearm using an EinScan PRO HD SHINING 3D scanner. Point image.

In the second stage of this study, a scan of the hand and part of the forearm was performed using an EinScan PRO HD SHINING 3D scanner. A point image was generated. The scan was performed in order to accurately fit the prosthesis to the potential patient’s hand so that the prosthesis would be comfortable to use (Figure 3).

### 3.3. Design Using CAD Software

**Figure 4:**
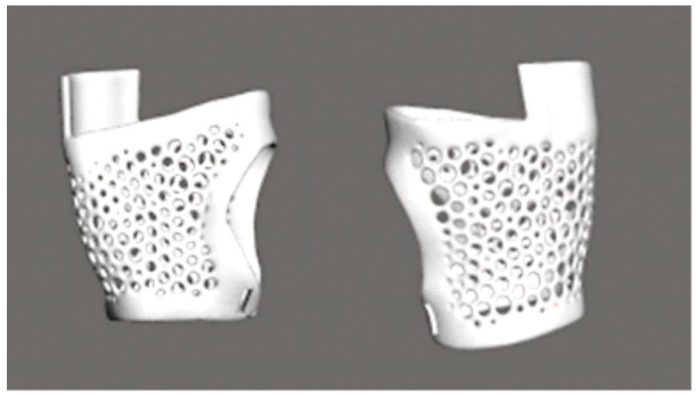
Part of the prosthesis that fits the hand has an openwork structure resembling a honeycomb, designed in MESH MIXER.

It is evident from the results of the analysis of the scan of the hand and forearm that the part of the prosthesis that adheres to the hand was fabricated using the “Make Pattern” function with the “Dual Edges” option in MESHMIXER program (Figure 4). This enabled the creation of an openwork structure based on a honeycomb motif. The prosthesis was designed to incorporate structural perforations in both the inner and outer surfaces, with the purpose of facilitating ventilation and reducing the overall weight of the component. These perforations also serve to minimize the strain on the user’s hand. In MESHMIXER program, the wall thickness and hole diameters were selected on the basis of experimental findings. In addition, all edges were rounded with a view to reducing skin irritation and enhancing the level of comfort experienced by the user during everyday use [25].

**Figure 5:**
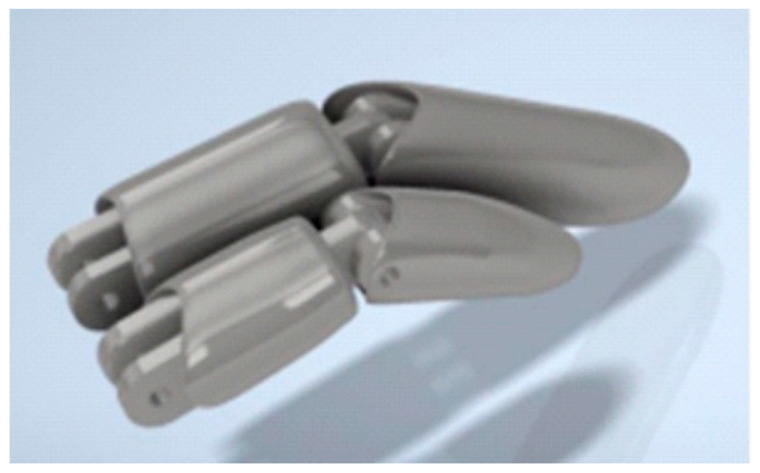
Model of missing parts of fingers based on the dimensions and shape of a human hand, designed in AUTODESK Inventor 2025.

Subsequently, a model of the finger parts was created based on the dimensions and shape of the human hand, taking into account the anatomical fit. This was designed in AUTODESK Inventor 2025 (Figure 5). The modelling process incorporated the incorporation of perforations on the phalangeal joints, which subsequently facilitated the subsequent mounting of pins. Great care was taken to ensure the precise replication of the smooth movement of the fingers. The modelling was performed using the solid method, employing the fundamental operations available in AUTODESK Inventor, in the following stages [16]:

– The creation of a base solid in the form of a cuboid with rounded edges is essential for the representation of the general cross-section and shape of the finger.
– The initial step in the procedure is the division of the solid into segments corresponding to individual phalanges. This approach facilitates the subsequent planning of the bending mechanism.
– The initial solid shape is modified through a series of processes involving cutting, rounding, and forming. These modifications are executed in a manner that emulates the technique of modelling from plasticine. The objective of this approach is to achieve a shape that is more natural and ergonomically designed.
– The utilization of the symmetry function was instrumental in ensuring the uniformity of parameters on both sides of the finger. This approach led to a substantial augmentation in the design’s velocity and precision.
– The process of cutting involves the creation of recesses, holes, and additional construction details.
– The following text is intended to provide a comprehensive overview of the subject matter.

**Figure 6:**
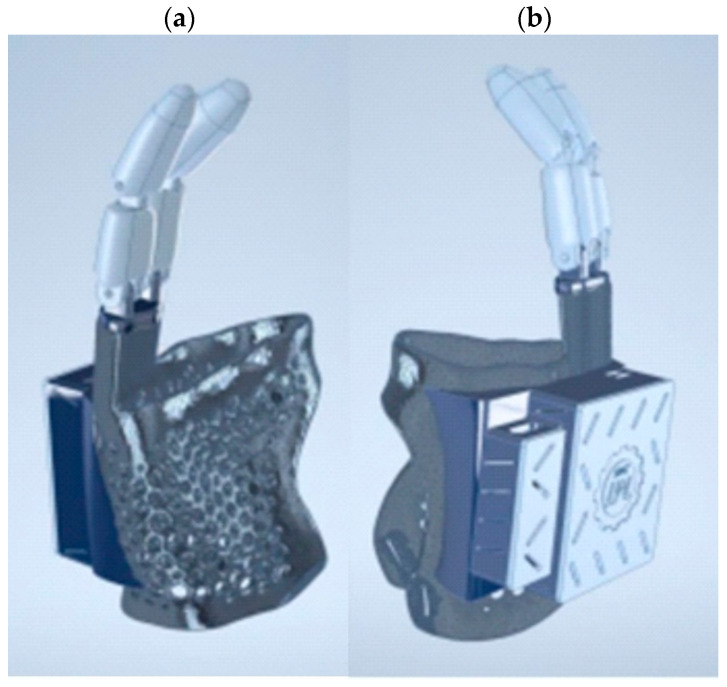
Visualization of the complex model of the prosthesis, connection of the phalanges with the prosthesis, and the box: (**a**) the inside of the prosthesis and (**b**) the outside of the prosthesis.

The connection of the phalanges with the prosthesis and the boxes allowed for precise visualization of the prosthesis (Figure 6).

### 3.4. Selection of Material

Careful selection of the material was made to ensure that it is both durable and safe for the patient’s skin. Important characteristics of prosthetic materials include strength, lightness, and comfort. Thus, polymeric materials are often used in this field. After analyzing various 3D printer filaments, a PLA filament was chosen.

### 3.5. Three-Dimensional Printing

Three-dimensional printing is a technology that is distinguished by both its flexibility and the ease with which it can undergo modification. The majority of models can be customized to suit individual requirements, both in terms of dimensions and functionality. This facilitates the design and implementation of even highly complex structures that are challenging or even unfeasible to produce using conventional methods [17]. A significant benefit of 3D printing is its rapid production speed. The process does not necessitate extensive planning or the creation of molds or tools, which are often time-consuming. In comparison with conventional methods, 3D printing has been shown to be both faster and more cost-effective, particularly in cases of individual or short-series production [25]. It is evident that three-dimensional printing is a more environmentally sustainable technology in comparison with a multitude of conventional production methodologies. This innovative technique enables the reduction in material waste by virtue of its ability to produce only what is required, in a layer-by-layer fashion. Moreover, the potential for the recycling of certain materials, in conjunction with a printing model that is exclusively order-based, serves to mitigate the environmental impact of the process [25].

**Figure 7:**
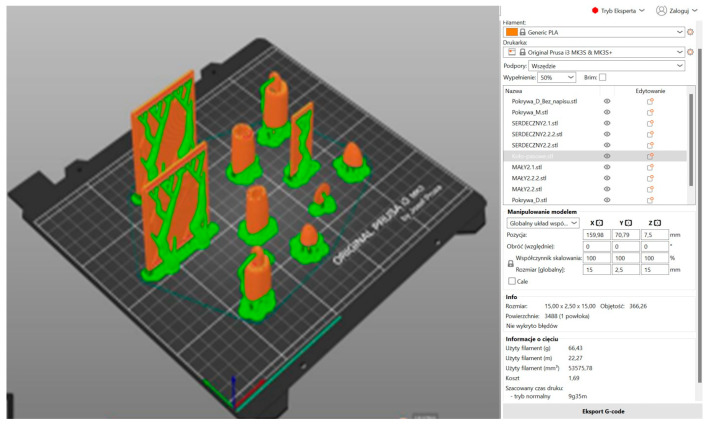
Three-dimensional printing model of the fingers and the box cover prepared in Prusa Slicer 2.8.1.

**Figure 8:**
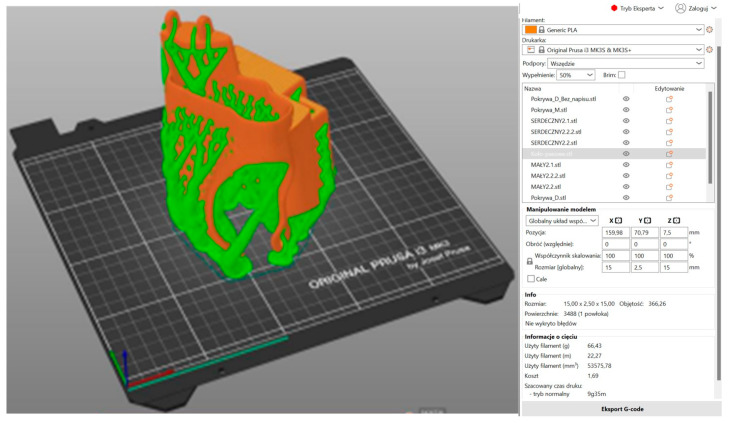
Three-dimensional printing model of the prosthesis body with boxes prepared in Prusa Slicer 2.8.1 (side view).

**Figure 9:**
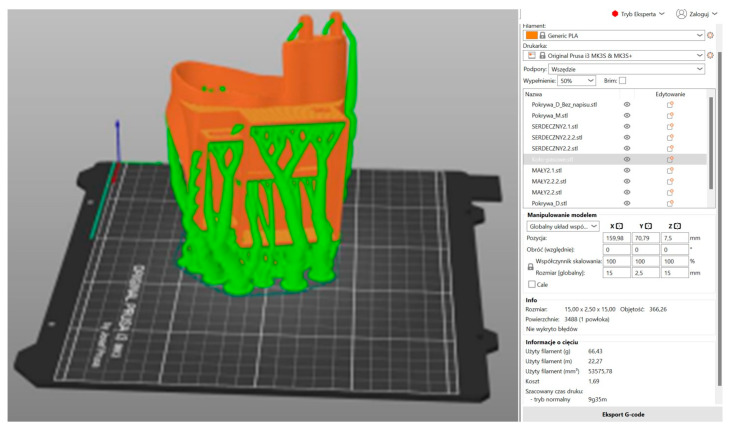
Three-dimensional printing model of the body of the prosthesis together with the boxes prepared in Prusa Slicer 2.8.1 (front view).

In the subsequent stage, the prosthesis model was prepared for 3D printing in Prusa Slicer 2.8.1 (Figure 7, Figure 8 and Figure 9). The utilization of 3D printing technology holds considerable promise in the fabrication of functional partial finger prostheses, thereby enhancing the functionality of individuals who have undergone amputation [4]. The organic support method, also referred to as the ‘tree method’, was utilized in the preparation of the fingers, body, and box covers for printing due to its ease of removal. In the initial phase, a comprehensive analysis of the model’s orientation on the build platform was conducted, with the components positioned in a manner that minimized the reliance on supports in critical areas. This approach served to mitigate the risk of surface defects. The individual printing parameters for each element were determined, including layer height (0.2 mm), print speed (approximately 50 mm/s), and nozzle temperature (200 °C). This ensured an optimal balance between the model’s level of detail and the time taken for printing. The utilization of a PLA filament was determined by its optimal adhesion to the build plate, minimal shrinkage, and biocompatibility during the prototyping stage [9,19]. It is also important to note that particular attention was paid to the appropriate setting of the heating table temperature (60 °C). This was carried out in order to ensure the stability of the first layers and to avoid the model peeling off during printing. Furthermore, during the project’s preparation phase, the ergonomics of the prosthesis and its target mechanical loads were given full consideration. It is evident that a higher filling density for smaller elements, such as fingers and covers, ensures their increased stiffness and resistance to damage during use. Consequently, the diminished volume of the prosthesis resulted in a substantial decrease in its mass, while preserving adequate strength. This is crucial from the perspective of the patient’s experience with the prosthesis during daily activities. In conclusion, the selection of 3D printing parameters enabled the optimization of material consumption and production time. Furthermore, it facilitated the creation of a functional prototype that fulfils the structural and functional requirements. This prototype can then be subjected to further testing and modification as part of the medical design process. The fabrication of the fingers and box cover involved the utilization of 50% filling and 66.43 g of PLA.

### 3.6. Design and Simulation of an Electronic Circuit

**Figure 10:**
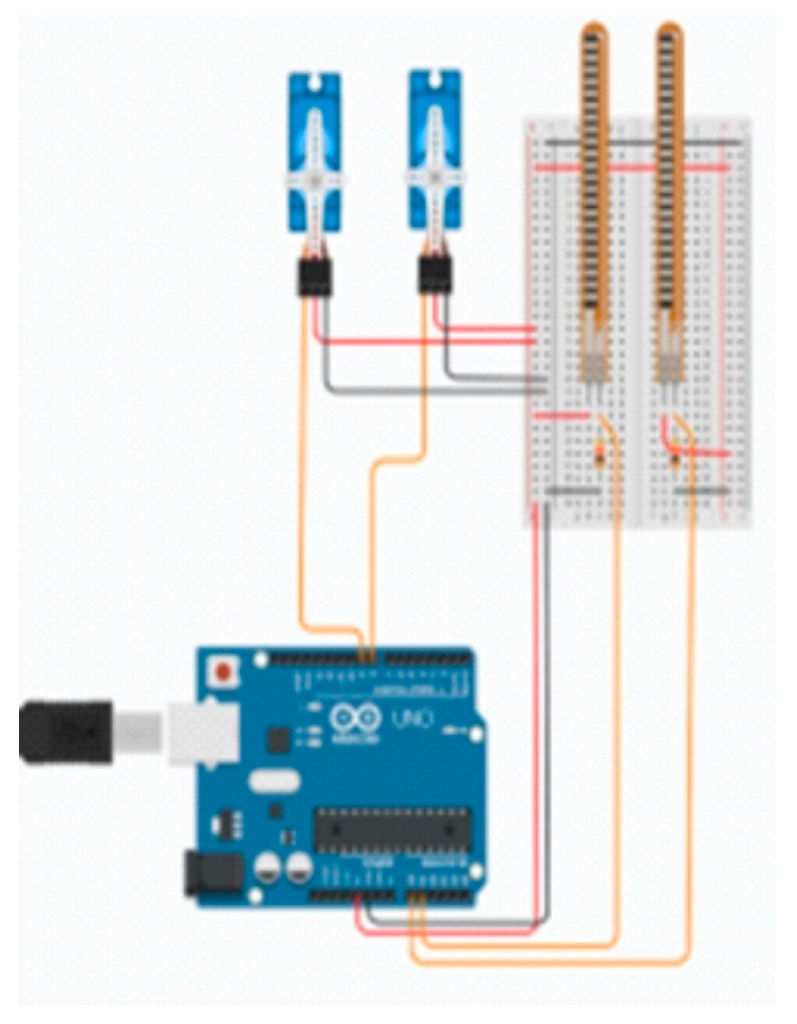
The design of the control system made using TinkerCad software.

The design of the control system was created using TinkerCad software, which facilitates the modelling of the system without the necessity of possessing individual components in physical form, thereby eliminating superfluous costs (Figure 10). TinkerCad program has been found to be an intuitive one, with a database of components and sample systems that present correct methods of assembling individual elements. The connection of components to the contact board may be facilitated by dragging them to the relevant location. The electronic system controlling the operation of the entire hand was based on basic electronic components, compatible with the Arduino Nano Every controller. The wires were connected to two bend sensors, with dimensions of 73 × 6.3 mm (SparkFun SEN-10264, SparkFun Electronics, Niwot, Colorado, USA, and two servos capable of 180° rotation. These components were then soldered to the controller, which was powered by a 5 V Feetech Wing FT3325 battery (Feetech, Shenzhen, Guangdong, China). The Arduino Nano Every controller (ABX00028) (Arduino, Scarmagno, Italy) is equipped with an ATmega4809 microcontroller (8-bit AVR) and has 48 kB Flash memory, 6 kB SRAM, and 256 B EEPROM. Power is supplied via the USB port (5 V) or the VIN pin (6–21 V). The system is equipped with 14 digital I/O pins (6 of which are PWM-capable) and 8 analog inputs, in addition to supporting UART, SPI, and I2C interfaces. The programming of the device is facilitated by the utilization of the Arduino IDE environment via a USB micro-B connector. The dimensions of the board are 45 × 18 mm. The bend sensors employed function as flexible resistive sensors, whereby they undergo a change in resistance in accordance with the degree of deflection. In a straight state, the resistance is approximately 10 kΩ; however, with significant bending, this can increase to between 60 and 110 kΩ. The bending angle of the fingers is determined based on the changes in resistance. Subsequent to the conversion of the angle value to degrees by the Arduino controller, the signal is transmitted to the servos. Subsequently, the servos rotate at the calculated angle, resulting in the tightening of the cables responsible for the movement of individual fingers. The system utilizes digital Feetech Wing FT3325 micro-servos, which are distinguished by their high positioning precision and rapid response time, attributable to digital control. The supply voltage range is from 4.8 to 6 V, with the torque reaching 5.85 kg/cm at a lower voltage and 7.21 kg/cm at 6 V. The rotation time of 60° is measured at 0.16 s at 4.8 V and 0.13 s at 6 V. The movement range of the servo is from 0 to 120°. The design is founded upon metal gears and double ball bearings, thus increasing durability and reliability. The dimensions of the servo are precisely 30 × 10 × 35.5 mm, and its weight is 26.2 g. These parameters allow for precise reproduction of calculated deflection angles and stable operation in systems requiring precise motion control. The sensors were connected to the analog pin, and the pins to the servos to enable control. Two motors were developed, with one sensor assigned to the first servo and the other to the second servo. Subsequently, the deflection angle was converted to a rotation angle, enabling the adjustment of the finger bending angle via a cable. Subsequent to the activation of the entire system, the servos were automatically adjusted to the 0° position. Following the conclusion of the program, a time delay was incorporated with a view to ensuring the creation of the most seamless system possible. The power supply unit (PSU) was utilized to facilitate the operation of the control system, thereby ensuring the attainment of a consistent 5 V voltage, a prerequisite for the effective functioning of all electronic components. This solution obviated the necessity for more complex power supply systems or voltage converters, while ensuring mobility and convenience of use. The utilization of a power bank facilitated the uncomplicated charging and replacement of the power source, obviating the need for interference with the device’s construction. The operational longevity of the system is contingent upon the battery capacity employed; in the event of a sufficiently high capacity, the system is capable of continuously functioning for a number of hours, rendering it suitable for a variety of applications, including functional tests, presentations, and educational use.

The program allows for the simulation of program operation, which was used when writing the code. This prevented the physical controller from being exposed, made it easier to check and correct the code through the program’s built-in error detection functions, and ultimately effectively speeded up work on the entire control system.

### 3.7. Assembly and Integration of the Electronic System into the Prosthesis

**Figure 11:**
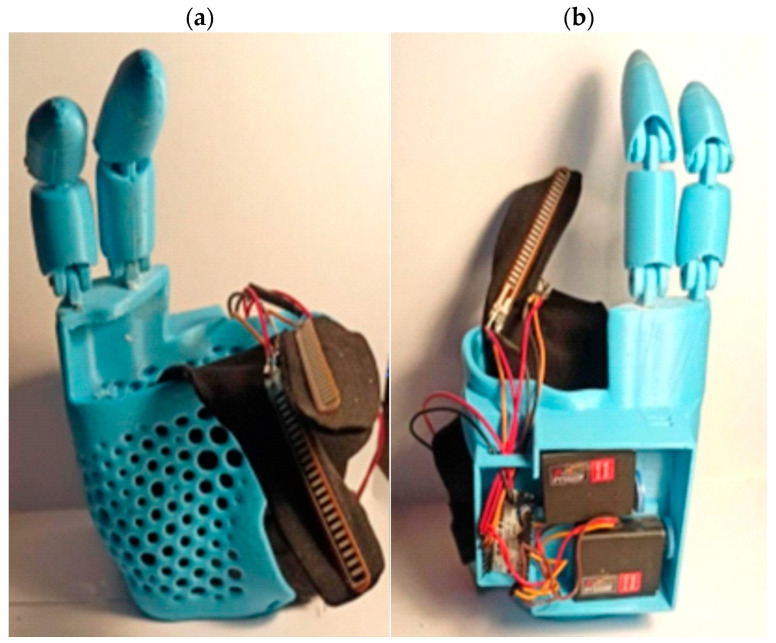
The final product of the PLA prosthesis (**a**) equipped with a control system and billiard glove (**b**).

In the final stage of prosthesis construction, the mechanical and electronic parts were assembled into a finished prosthesis, which enabled testing and functional analysis. The electronics were soldered to an Arduino board, the motors were placed in dedicated boxes, and the sensors were sewn onto a billiard glove, allowing the prosthesis to be controlled using the fingers. From the tips of the printed fingers, fishing lines (two for each finger) were led up to the motors, which are responsible for the bending functions of these fingers. The 3D-printed parts were connected by pins made of steel, which were fixed into the prosthesis. Once the prosthesis was assembled, a program was uploaded to test the operation of the entire system (Figure 11).

In [27], the design and testing of an electromechanical artificial finger prosthesis driven by two dielectric elastomer actuators (DEAs) arranged as agonist–antagonist pairs as artificial muscles was presented. The prosthesis model incorporated bending motion utilizing a single pair of DEAs. The prosthesis was fabricated from PLA using 3D FDM technology, with the objective of achieving lightweight construction.

## 4. Conclusions

The present work sets out the processes involved in the design, modelling, and manufacture of a right-handed prosthesis, with the fabrication undertaken using a PLA material. The results of this study confirmed that it is possible to produce a functional and patient-specific hand prosthesis. The prosthesis was conceived as a straightforward and cost-effective solution, intended for utilization by individuals living with disabilities. It was hypothesized that popular, inexpensive components and materials readily available on the market are utilized. All software packages utilized in this study were either freely available or obtained under a university license, thus incurring no additional expenses. Operating costs associated with the 3D printer, scanner, and electricity consumption were excluded from the analysis, given that the printing was conducted on the university premises as part of a PBL (Project Based Learning) initiative. The total cost of the prosthesis did not exceed PLN 1000 (~EUR 230).

The use of 3D printing technology and a simple Arduino-based electronic circuit meant that the assumptions regarding the comfort and safety of the patient’s hands were met. It was concluded that the prosthesis can be developed as a viable solution for people with mobility impairments, including those with partial amputations. Simulations in TinkerCAD enabled errors to be detected earlier, thereby improving the integration of mechanics and electronics. The simulation showed that the finger control system worked as intended. Using 3D printing technology and readily available materials increases the product’s availability to a larger potential audience.

The fabricated prosthesis allows for the further development of this type of product, for example by using more advanced materials or improving the control mechanisms.

## Data Availability

The original contributions presented in this study are included in the article. Further inquiries can be directed to the corresponding author.
